# Electrical Therapy for Heart Failure: The Year 2023 in Review

**DOI:** 10.19102/icrm.2024.15018

**Published:** 2024-01-15

**Authors:** Imran Niazi

**Affiliations:** 1 Aurora Cardiovascular and Thoracic Services, Aurora Sinai/Aurora St. Luke’s Medical Centers, University of Wisconsin School of Medicine and Public Health, University of Wisconsin–Madison, Madison, WI, USA

**Keywords:** Cardiac contractility modulation, cardiac resynchronization therapy, conduction system pacing, heart failure

This past year, physician-scientists continued to advance our understanding of pacing and related therapies for heart failure. Evidence for the efficacy of conduction system pacing (CSP) continued to accumulate, although large randomized trials, such as those supporting cardiac resynchronization therapy (CRT), are still lacking. Important clinical questions remain: which patients indicated for CRT should receive CSP, and when should CRT be used? Should both pacing modalities be used in select patients?

There are recognized limits intrinsic to each therapy; CRT is limited by anatomy, in that targeted pacing of late-activated segments of the left ventricle is not always achievable, and the pacing stimulus is necessarily delivered to the epicardium. With CSP, a significant proportion of patients with heart failure have delayed conduction distal to the pacing site, which hinders intra-left ventricular (LV) synchrony.^1^ Applying both therapies in the same patient promises to overcome the shortcomings of each therapy, but at an additional cost, and it may not be necessary in every patient.

Vijayaraman et al. attempted to answer this question with a randomized prospective pilot study based on an intriguing concept: pacing the proximal His–Purkinje system (HPS) may allow us to select the patients without significant delayed distal conduction.^2^ Such patients can receive CSP, while those exhibiting distal delayed conduction can receive a CRT lead in addition to CSP. In their three-center, single-blind study, 100 consecutive patients meeting the standard CRT criteria were randomized in a 1:1 fashion to HPS CSP-optimized CRT (HOT CRT) or conventional biventricular pacing CRT (BVP CRT). In the HOT CRT group, 4 patients received corrective His-bundle pacing; 39 received left bundle branch area pacing (LBBAP); and 5 received left septal pacing plus a CRT lead, as corrective LBBAP could not be achieved. The remaining two patients crossed over to the biventricular (BIV) pacing arm. In the BVP CRT group, 41 patients successfully received BIV pacing, while 9 patients who failed crossed over to CSP. There was a statistically significant greater improvement in the left ventricular ejection fraction (LVEF) at 6 months in the HOT CRT cohort compared to the BVP CRT cohort (12.4% ± 7.3% vs. 8.0% ± 10.1%, *P* = .02). There was no difference in clinical endpoints such as mortality or hospitalizations for heart failure, but this pilot study did not have a sufficient number of patients to detect such differences.

At first blush, an LVEF improvement of 8% or 12% does not seem clinically remarkable. In a patient with a baseline LVEF of 30%, the EF would improve to 32.6% and 33.6%, respectively. We recall though that similar improvements in LVEF were associated with quite striking improvements in survival in the landmark CRT studies. For example, in the Multicenter Automatic Defibrillator Implantation Trial with Cardiac Resynchronization Therapy (MADIT-CRT), the improvement in LVEF was 8% at 1 year.^3^ Larger controlled trials are required to clarify the clinical and mortality benefits with the various CSP strategies.

It is also worthwhile to consider the impact of an additional proximal septal lead on tricuspid valve function over time. The present implant techniques do not emphasize placing the septal lead for LBBAP beyond the septal leaflet of the tricuspid valve or in the commissure between the anterior and septal tricuspid leaflets.

Hu et al. reported on the impact of left bundle pacing leads on tricuspid valve function earlier this year.^4^ They studied 89 patients who underwent LBBAP for currently accepted indications. Echocardiography was used to quantify the severity of tricuspid regurgitation (TR) and LBBAP lead location. Patients were classified into two groups: 58 had trivial or no TR and 31 had moderate-to-severe TR on the preprocedural echocardiogram. At 19 ± 6.5 months following implant, TR had developed or worsened in 23 patients (39%) in the first group and worsened in 6 patients (19%) in the second group. Overall, 32.5% of patients undergoing LBBAP developed new TR or had worsening of the previous TR. In patients with trivial or no TR, a mean distance of ≥16.1 mm between the lead fixation point and the tricuspid annulus was an independent predictor of new or worse TR. Larger long-term studies will be needed to assess this impact. Coronary sinus leads for BIV CRT do not cross the tricuspid valve, which is a potential benefit. Time will tell if this translates into a clinical advantage.

Many experts advise placing the LBBAP lead 10–20 mm from the His-bundle location. This is well within the area shown by Hu et al. to impact the tricuspid valve function. Perhaps it is time to reconsider this recommendation. We routinely image the tricuspid valve leaflets by contrast injection and take care to place the LBBAP lead beyond their limits.

Another leadless solution for CRT is the WiSE-CRT (for Wireless Stimulation Endocardially for Cardiac Resynchronization) system (EBR Systems, Sunnyvale, CA, USA), which completed its pivotal trial (Stimulation of the Left Ventricular Endocardium for Cardiac Resynchronization Therapy [SOLVE-CRT]) in 2023 and is undergoing evaluation by the U.S. Food and Drug Administration (FDA) for clinical approval at this time. This system consists of a subcutaneous ultrasound transmitter/pulse generator and a percutaneously delivered LV endocardial receiver electrode about the size of a grain of rice. Ultrasound pulses timed to coincide with a right ventricular (RV) pacing stimulus induce an electrical current in the endocardial electrode, initiating LV contraction. Endocardial LV stimulation is arguably more physiologic than conventional CRT and has the potential to improve patients who fail to respond to conventional CRT. The ALternate Site Cardiac ResYNChronization (ALSYNCH) study^5^ demonstrated a 47% response rate in CRT non-responders, and the SOLVE-CRT trial did include a cohort of such patients. The results of SOLVE-CRT are embargoed pending publication at this time.

The WiSE-CRT system does offer the opportunity for a completely leadless system. While most cases involve implantation with a conventional transvenous RV lead for RV pacing, it can be paired with a leadless RV pacemaker **(Figure 1)**. Carabelli et al.^6^ reported eight cases with concomitant Micra™ leadless pacemaker (Medtronic, Minneapolis, MN, USA) and WiSE-CRT implant. All implants were successful at first attempt. The WiSE-CRT system has four sensing electrodes on the transmitter, which are used to detect the RV pacing output of the co-implanted Micra™ pacing device. Detection of the RV pacing spike triggers the WiSE-CRT system to deliver its focused ultrasound beam toward the receiving electrode, thereby initiating LV stimulation coincident with RV stimulation. In this group, WiSE-CRT improved electrical dyssynchrony (paced QRS, 204.38 ± 30.26 vs. 137.5 ± 24.75 mS; *P* = .012) and also LVEF (28.43% ± 8.01% vs. 39.71% ± 11.89%, *P* = .018), with a 41% increase following 6 months of therapy.

Earlier this year, cardiac contractility modulation (CCM) with the Optimizer (Impulse Dynamics, Marlton, NJ, USA) gained U.S. FDA approval for use in patients with symptomatic heart failure and a mild-to-moderate reduction in LVEF (25%–40%). CCM differs from the various forms of pacing-mediated cardiac synchronization discussed already, as it is effective in narrow QRS patients without electrical dyssynchrony and helps the failing heart by different mechanisms. The device consists of a rechargeable generator, usually implanted in the right infraclavicular fossa, which connects to the RV septum via two transvenous screw-in electrodes for the delivery of non-excitatory pulses delivered in the ventricular refractory period **(Figure 2)**. CCM treatment reverses the cardiac maladaptive fetal gene program seen in heart failure and normalizes the expression of key sarcoplasmic reticulum Ca^2+^ cycling and stretch response genes.^7^ A large body of evidence supports the use of CCM for improving symptoms, exercise tolerance, and heart failure admissions in heart failure patients.^8^ Longer-term follow-up in registries has also demonstrated improvements in LVEF, heart failure hospitalizations, and survival compared to historical controls.^9^ **Figure 2** illustrates the typical disposition of this device in a patient with a concomitant implantable cardioverter-defibrillator. This arrangement necessitates two separate devices, possibly compromising venous access on both sides. To overcome this, a combined device capable of pacing, defibrillation, and CCM has been developed and is presently undergoing evaluation in clinical trials.

## Figures and Tables

**Figure 1:**
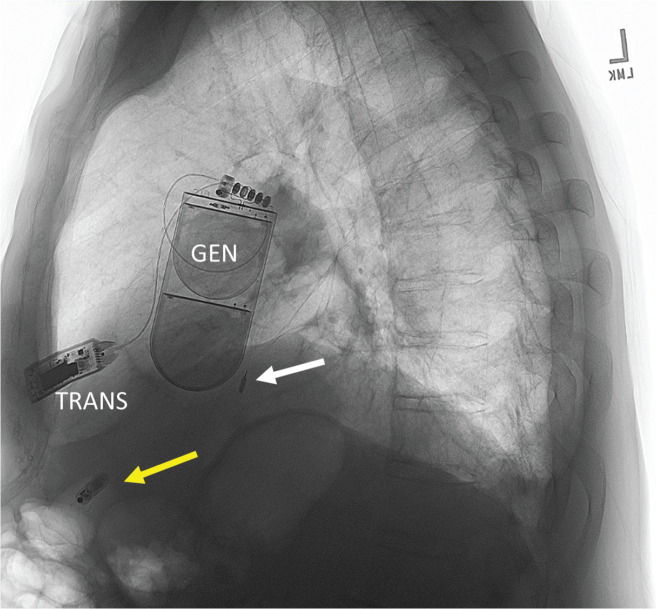
A completely leadless biventricular pacing system involving WiSE-CRT plus Micra™. A white arrow indicates the left ventricular endocardial electrode, while the yellow arrow indicates the Micra™ leadless right ventricular pacemaker. *Abbreviations:* GEN, EBR Systems generator; TRANS, subcutaneous focused ultrasound transmitter.

**Figure 2:**
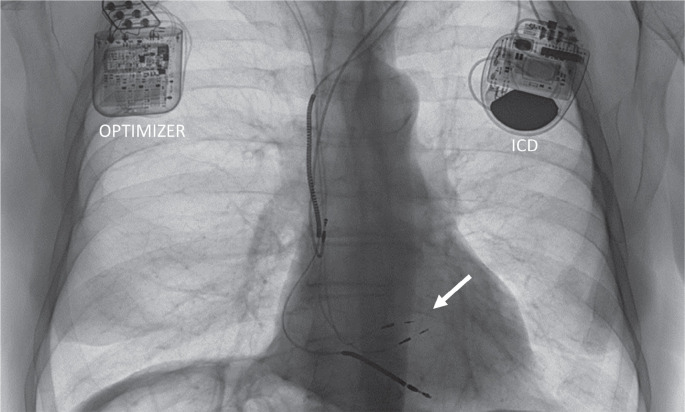
The Optimizer generator in the right subclavian fossa; the arrow points to two leads screwed into the mid-septum. An implantable cardioverter-defibrillator (ICD) generator (left chest) and right ventricular ICD lead are present in the right ventricular apex. *Abbreviation:* ICD, implantable cardioverter-defibrillator.
